# Meat Safety in Tanzania’s Value Chain: Experiences, Explanations and Expectations in Butcheries and Eateries

**DOI:** 10.3390/ijerph17082833

**Published:** 2020-04-20

**Authors:** Gerard Prinsen, Jackie Benschop, Sarah Cleaveland, John A. Crump, Nigel P. French, Tabitha A. Hrynick, Boniface Mariki, Blandina T. Mmbaga, Joanne P. Sharp, Emmanuel S. Swai, Kate M. Thomas, Ruth N. Zadoks, Linda Waldman

**Affiliations:** 1 School of People, Environment and Planning, Massey University, Bag 11222, Palmerston North 4442, New Zealand; 2 School of Veterinary Science, Massey University, Bag 11222, Palmerston North 4442, New Zealand; j.benschop@massey.ac.nz (J.B.); n.p.french@massey.ac.nz (N.P.F.); 3 Institute of Biodiversity, Animal Health and Comparative Medicine, College of Medical, Veterinary and Life Sciences, University of Glasgow, Glasgow G12 8QQ, UK; sarah.cleaveland@glasgow.ac.uk (S.C.); ruth.zadoks@sydney.edu.au (R.N.Z.); 4 Centre for International Health, University of Otago, PO Box 56, Dunedin 9054, New Zealand; john.crump@otago.ac.nz (J.A.C.); k.thomas@otago.ac.nz (K.M.T.); 5 Institute of Development Studies, University of Sussex, Library Road, Brighton BN1 9RE, UK; T.Hrynick1@ids.ac.uk (T.A.H.); L.Waldman@ids.ac.uk (L.W.); 6 Tanzania Chamber of Commerce Kilimanjaro, Old Moshi Road, Moshi 9713, Tanzania; marikiboniface20@gmail.com; 7 Kilimanjaro Christian Medical University College, Moshi 2044, Tanzania; blaymt@gmail.com; 8 School of Geography and Sustainable Development, University of St Andrews, St Andrews KY16 9AL, UK; js314@st-andrews.ac.uk; 9 Ministry of Livestock and Fisheries, Dodoma, PO Box 2870, Tanzania; esswai@gmail.com; 10 Sydney School of Veterinary Science, University of Sydney, JL Shute Building, Camden, NSW 2570, Australia

**Keywords:** red meat, food safety, value chains, butcheries, eateries, Tanzania

## Abstract

Urbanisation is associated with changes in consumption patterns and food production processes. These patterns and processes can increase or decrease the risks of outbreaks of foodborne diseases and are generally accompanied by changes in food safety policies and regulations about food handling. This affects consumers, as well as people economically engaged in the food value chain. This study looks at Tanzania’s red meat value chain—which in its totality involves about one third of the population—and focuses on the knowledge, attitudes and reported practices of operators of butcheries and eateries with regards to meat safety in an urban and in a rural environment. We interviewed 64 operators about their experiences with foodborne diseases and their explanations and expectations around meat safety, with a particular emphasis on how they understood their own actions regarding food safety risks vis-à-vis regulations. We found operators of eateries emphasising their own agency in keeping meat safe, whereas operators of butcheries—whose products are more closely inspected—relied more on official inspections. Looking towards meat safety in the future, interviewees in rural areas were, relative to their urban counterparts, more optimistic, which we attribute to rural operators’ shorter and relatively unmediated value chains.

## 1. Introduction

Considerable academic and policy attention has focused on meat value chains and the production of meat safe for human consumption in East and Central Africa in recent years [1,2,3,4,5,6,7]. Concern over meat production is driven, in part, by urbanisation, changing lifestyles, increased incomes, and changing dietary practices [8,9] and, in part, by the presence of endemic zoonotic diseases. (The term “zoonoses” refers to “any disease or infection caused by all types of agents, bacteria, parasites, fungi, viruses and unconventional agents, transmissible from vertebrate animals to humans and *vice-versa*” [10] (p. 77).) This combination can have negative impacts on people’s health and livelihoods [11] as intensification of production, central processing, and large-scale distribution can place many people at risk from the same source of infection. Conversely, intensification may offer new opportunities for improved food safety practices, as demonstrated for major zoonotic foodborne pathogens such as non-typhoidal *Salmonella* in the egg supply chain in the UK [12], *Campylobacter* in the poultry supply chain in New Zealand [13], and verotoxin-producing *Escherichia coli* in the beef supply chain in Canada [14]. In all these examples, food safety policies, regulations, industry-led changes in red meat processing and public education play a role in improving meat safety. In this paper we examine the pre-consumer links of Tanzania’s red meat value chain, namely the butcheries selling raw meat and the eateries selling cooked meat to consumers. In particular, we explore meat-sellers’ experiences, expectations, and explanations of meat safety and zoonoses. We investigate their practical constraints and their personal agency (i.e., their views on food safety and on how their subsequent handling of meat affects food safety risks risks), and rural–urban differences in relation to meat safety in Tanzania.

Tanzania’s red meat value chain includes the raising of sheep, goats and cattle, transportation, slaughter and butchering of animals, transporting meat, the processing of meat products, and by-products for local consumption and export. It is ”estimated that up to one third of the population in one way or another is engaged in the production, processing, and selling of red meat” [3] (p. 12). This includes livestock owners, market agents, dealers, animal transporters, inspectors, butchers, processors, researchers, extension workers, wage labourers, exporters and importers, meat-sellers, eateries, restaurants, and supermarkets [2].

The livestock industry (meat, dairy, and poultry) makes a substantial contribution to Tanzania’s economy [15]. In 2016/17 Tanzania produced almost 500,000 tonnes of red meat, 85% of which came from cattle [16]. Cattle producers tend to be small-scale mixed commercial farmers, agro-pastoralists, or pastoralists. The latter may have large herds, yet rely on traditional livestock-keeping practices and indigenous cattle rather than modern farming methods, intensification and “improved” stock. Cattle bought and sold at primary, secondary and border markets are slaughtered at slaughter slabs in rural areas and peripheral urban wards, and slaughterhouses and abattoirs in urban areas. Slabs are generally very basic concrete pads which occasionally have corner posts supporting a roof. Slaughterhouses are larger, enclosed buildings operating formally with some infrastructure (drainage systems and running water) and equipment (hooks). Slaughterhouses are, however, less mechanized than formal abattoirs [17]. Raw meat-sellers, or butchers, sell this “warm” meat, *nyama kawaida*, or “undifferentiated meat with or without bones” [2] (p. 7) with little added value. This satisfies rural and urban households’ demand for meat [18,19].

Tanzania’s butchers are required to meet infrastructural and hygiene requirements, and are inspected to ensure they comply with food safety standards [20], although in practice this involves a complicated combination of pre-arranged as well as impromptu inspection, negotiation, diplomacy, self-regulation, and occasional clamp-down [21]. Operators of eateries cook raw meat purchased from butchers and prepare meat soup (*supu*) and/or roasted meat or *nyama choma* for their customers. These cooked meat-sellers’ facilities are usually rather basic, comprising of small cooking areas and plastic seating or wooden benches where customers eat. These eateries, like the butcheries, are regulated and inspected by Health Officers.

The growing attention to food safety and foodborne diseases in low- and middle-income countries (LMICs)—defined as “any disease that results from the ingestion of contaminated or naturally hazardous foods” [22] (p. 10491)—focuses on the increasing globalisation of food production; the lengthening and intensification of agri-food value chains; the advent of new technologies; consumer demand for safe, fresh, high quality food; increasing urbanisation; and foreign investment. While much foodborne disease remains underreported and undetected, research in Tanzania seems to have emphasised food safety in the dairy and fish sectors [23,24,25,26] and in consumer preferences [20,27,28]. In terms of red meat and food safety, the Tanzanian government, in keeping with many other East African countries, has recognised that Tanzanian citizens are potentially exposed to pathogens that may cause zoonotic diseases via meat or other foodborne transmission, including but not limited to bovine tuberculosis, brucellosis, anthrax, salmonellosis, and campylobacteriosis [1,9,26,29,30].

Investigating knowledge, attitudes, and practices (KAP) of workers handling meat prior to consumption—i.e., in butcheries and eateries—and their roles in spreading or containing infectious organisms in LMICs comprises an important focus in red meat food safety research [31,32,33,34]. The literature also details activities which undermine food safety—such as eating raw and under-cooked meat, eating meat from sick animals, putting fresh blood on day-old meat [5], workers’ lack of or poor use of hygienic and protective equipment, working when ill, inappropriate footwear, unhygienic handling of animal products, and lack of training [35,36]. Often, the emphasis is on what butchers and other actors know about food-borne diseases in scientific terms, which zoonoses they can name, whether they can expand on clinical symptoms, and how they assess the effect of their practices on meat safety [33].

In the specific context of Tanzania, several studies have reported on the low levels of zoonotic disease knowledge, and consequent attitudes and practices of livestock keepers, animal health workers, and health personnel [30,37,38,39]. Most livestock production takes place in rural and peri-urban areas, where producers experience challenges when they seek to maximise value chain opportunities. Most ‘traditional’ retail outlets and markets serve rural and peri-urban citizens [27]. However, at the same time Tanzania’s cities are growing and urbanisation has resulted in some rural or peri-urban areas being reclassified as urban areas [40]. This urbanisation has been accompanied by economic growth, changing dietary preferences, and the emergence of supermarkets with their own supply chains throughout Tanzania as much as elsewhere in East Africa [41].

In this paper, we draw on data collected during a multidisciplinary and transnational research project between 2014–2018 to investigate Hazards Associated with Zoonotic enteric pathogens in Emerging Livestock meat pathways (HAZEL). The project was led by the University of Glasgow, in close cooperation with Tanzanian research institutes. It comprised a broad field of research activities, ranging from meat value chain mapping to molecular epidemiology and mathematical modelling. Much of the research focused on the potential presence of zoonotic foodborne pathogens in meat and rural-urban transitions as a departure point. This paper focuses on the knowledge of meat safety held by people selling raw and cooked meat in, respectively, butcheries and eateries. However, instead of assessing this knowledge in relation to scientific knowledge or taking a KAP approach—which emphasises knowledge of particular disease transmission pathways and their impacts on food safety, characteristic of much research in this area—we adopt an emic approach which prioritises the perspectives and viewpoints of butchers and cooked meat-sellers. In so doing, the paper draws attention to meat-sellers’ meanings, sense of purpose, and their scope for undertaking or ensuring food safety. Next to individuals’ roles in the meat value chain, their sense of agency occurs in response to, and is shaped by, structural conditions such as institutionalisation, policies and regulations, and socio-economic and political circumstances [42], which can play out differently in rural or urban areas. We posit, as do other authors, that better understandings of meat-sellers’ own experiences and expectations can help to build incentives for positive behavioural change [8,43]. We also examine how meat-sellers perceive the future of food safety, drawing upon Høg and colleagues’ concept of the “temporalities of risk perception” [44] (p. 1), which argues that sociocultural contexts and past experiences affect the ways in which people perceive future risk, whereby “risk which is calculated retrospectively is unlikely to encourage investment in biosecurity” [44] (p. 2).

In our approach, the notion of agency is important. It attributes to individuals the capacity to understand and evaluate social experiences and acknowledges their scope for choosing to take particular actions, even in conditions limited by inadequate knowledge, coercion, or other constraints [45]. In other words, agency is a recognition of people’s own ability to resolve problems, to shape social events in particular ways, to monitor and reflect upon their own and others’ actions and an acknowledgement of constraining or enabling structures and circumstances [42]. The concept of agency also takes relationships into account, looking at how actors use and respond to networks of relationships and the flows of information, resources, and orders or regulations. We pay attention to individual operators’ actions and choices in relation to food safety in butcheries and eateries, the ways in which these are shaped through gender, prevailing discourse and interaction, and are limited by structural conditions in the rural or urban areas where actors reside.

## 2. Materials and Methods

This project received ethical approval from the Ethics Committee of the College of Medical, Veterinary and Life Sciences at the University of Glasgow, Glasgow, UK (Ref. 200,140,183 and Ref. 200140152); the Human Research Ethics Committee at the University of Otago, Dunedin, New Zealand (Ref. H15/069); the Tanzania’s National Institute of Medical Research (Ref. NIMR/HQ/R.8a/Vol. IX/2028 and extension Ref. NIMR/HQIR.8cNol. 11/1069); and the Kilimanjaro Christian Medical Centre (KCMC) Ethics Committee (Research Ethical Certificate No. 832). Permission to publish this paper was granted by the Director of Research, Information, Technology, and Communication of Tanzania’s National Institute of Medical Research on 28 February 2020 under reference NIMR/HQ/P.12VOLXXIX/115.

### 2.1. Sampling and Interviewing

We interviewed 64 people operating butcheries and eateries (most of whom were also the business owners) from March through to November 2016. The interviews were done in two of Tanzania’s administrative units: the urban area served by Moshi Municipal Council (called Moshi Urban hereafter) and the rural environment of Moshi District Council (Moshi Rural hereafter). Each unit comprises a number of lower administrative units called wards (31 wards in Moshi Rural and 21 in Moshi Urban). After undertaking four pilot interviews, the research team randomly selected five wards in each district. With the endorsement of district authorities, a project researcher approached local ward authorities for assistance in the identification of and introduction to butcheries and eateries—as required according to Tanzanian research protocol. The researchers asked to be introduced to people running licensed butcheries and eateries that were geographically widely distributed to avoid a “tarmac” bias [46] (p. 17).

After the introduction by local authorities, the interviewer and prospective interviewees agreed on a later time, when they met for the actual interview without the local official present. Interviewees gave recorded, verbal consent to participate, as explicitly endorsed by the ethical approvals obtained for this research. Considering available time and resources, three eateries and three butcheries in each of the five wards were approached for an interview, bringing the total number of interviews to 64. Compared to a Moshi Chamber of Commerce list of licensed butcheries, the sample represents about 5% of the butcheries operating in the two districts. We interviewed only eateries that were licensed; we did not include informal or roadside eateries serving food without a licence (*mama lishe* in Kiswahili). A list of licensed eateries could not be obtained. Disaggregation of data in our sample by gender shows that all butcheries were managed by men, whereas about one third of the eateries were managed by women (29% in Moshi Urban and 33% in Moshi Rural.)

The 64 interviews were conducted by a skilled Tanzanian interviewer, familiar with local political contexts and private sector businesses, who also undertook the transcription, translation and reviewing of interpretations and meanings. Interviews were guided by a 15-item semi-structured questionnaire and, with the consent of the interviewee, audio-recorded. (The questionnaire and the dataset created and analysed for this paper are available from the corresponding author on reasonable request.) The items covered a range of questions that were in a carefully calibrated way aligned with other HAZEL research being undertaken with actors in other sections of the meat value chain such as operators of slaughter slabs and frontline government officials [21,47]. Definitions of “major events,” “food safety,” etc. were neither provided nor requested, and were deliberately left open to enable people to answer in any way they saw fit and to draw on a range of interpretations. The interviews were conducted in Kiswahili, Kichagga, or Kipare, the most frequently spoken languages of the study area, with the choice of language left to each interviewee. After each interview, those parts of the recordings that had relevance to the questions were transcribed and, thereafter, translated into English by the interviewer. The interview responses were placed into a single document in which the answers of all interviewees were organised per question and then clustered in four groups: answers of urban butcheries, rural butcheries, urban eateries, and rural eateries. This facilitated a dual axes analysis; on the one hand comparing the answers of urban and the rural interviewees to look for patterns that were most likely driven by the environment of the businesses and, on the other hand, comparing the answers from butcheries with the answers from eateries to search for patterns typical of the type of business.

### 2.2. Data Analysis

The analysis of the answers was guided by two distinct approaches. First, we looked for patterns of recurring issues. This has been described as “inductive content analysis similar to a grounded theory approach” [48] (p. 420) in which preconceived coding categories are avoided and, instead, categories or patterns emerge from the raw data. For this research, a “pattern” is defined as something that is mentioned by at least three interviewees within one of the four groups of 15–17 interviewees. The minimum of three means at least 20% of the sample shows this pattern, which is deemed to make it more than just one or two individual interviewees’ views or experiences. This approach resulted in quantified patterns. Second, in a more qualitative approach described as “summative content analysis” [49] (p. 1283), we combed through the answers to all interview questions looking for recurring issues or explanations and considering issue-specific answers in the context of the entire interview, and in relation to answers given to other questions. The searching for patterns and the combing for recurring issues was done independently by two researchers and the patterns and recurring issues presented in this paper reflect overlap or consensus between these two researchers, creating a basis for intercoder reliability [50]. Conventional anthropological coding was used rather than data analysis software. The results we present in this paper are based on the analyses of these interviews and focus on respondents’ experiences of perceived major events or problems with meat safety, their views on why food safety incidents had occurred or were prevented, and their views on the safety of meat in the future. This data is differentiated by operational environment (urban or rural) and business type (butchery or eatery) and, where applicable, ranks respondents’ answers according to the frequency with which they are mentioned.

## 3. Results

### 3.1. Experiences in Butcheries and Eateries with Meat Safety Problems

After a series of introductions and discussions about their work, duties, and corresponding policies and regulations, operators of butcheries and operators of eateries in rural and urban Moshi were asked if they had ever, in all the years that they had been working with meat, experienced any major events or problems with meat safety. In both urban and rural areas, the majority of respondents (urban butchers 82%, rural butchers 87%; urban eateries 94% and rural eateries 93%) were emphatic that they, and their customers, had not experienced any major problems with food safety, whereby food safety was not actually defined.

*I have never experienced any kind of major event with meat safety.*

(Urban butchery)

*There is no major event that has occurred here. I am 100% sure.*

(Urban eatery)

Even the few interviewees who said they had experienced major events with meat safety were—close reading of their answers—referring to relatively recent incidents involving cases of a single cow or goat being declared diseased or unfit for sale. Two of the 64 interviewees referred to experiences with specifically named outbreaks of disease:

*There was an outbreak of Rift Valley Disease, I don’t remember when. We were forced to close the service for a week or so.*

(Urban butchery)

*Since inception, I have not come across such a bad incident. I have heard of outbreak of anthrax disease in Rombo District, but not here.*

(Urban butchery) (*A newspaper reported on this Anthrax outbreak in April 2016 [51]*)

No respondents recalled incidents in which their customers had reported back that meat sold had caused anyone to be taken ill, although one remembered hearing that another butcher had sold meat which, his customers believed, had caused a number of them to have diarrhoea. As discussed in the next section, they had particular reasons which explained why they had been able to avoid similar experiences.

### 3.2. Explanations for Meat-Sellers’ Confidence that Meat Is Safe to Consume

When asked about major problems with food safety, most (53 of the 64 or 83%) of the respondents continued, unprompted and encouraged as the interviews were semi-structured, to explain why this was the case. These explanations offer revealing insights into interviewees’ perceptions of responsibility and agency around meat safety. Our analysis of their explanations suggests they fall into two main categories. First, there are explanations that refer to preventative actions by external agents (hereafter referred to as external agency), mostly inspections by Veterinary Officers (VOs) and other regulators such as Livestock Extension Officers (LEOs) and Health Officers (HOs). Second, there are explanations that refer to their own preventative actions (e.g., keeping the premises clean, or boiling the meat for a long time, referred to hereafter as own agency). Table 1 provides a quantified analysis of this qualitative data, showing the distribution of the frequency of which the two types of agency were mentioned, with the added note that some interviewees, of course, mentioned both types of agency as an explanation.

This shows that, when external and personal agency as explanations for preventing major meat safety events are compared, urban and rural butcheries mention actions by external agents more often than their own actions. By contrast, operators of eateries (both urban and rural) have a marked belief in their own agency as the principal explanation for the prevention of food safety problems.

The following quotes exemplify urban and rural butchers’ perspectives, which emphasise both the role of external agents (external agency) and their own personal attention to food safety (own agency):

*This has not happened here, not only to me but to all butchers around. I think the reason is that the animal doctors [LEOs or VOs] are very keen here. We are also very eager in ensuring that business’ physical environment is clean and favourable. This ensures that the meat sold here is safe to eat.*

(Rural butchery)

*No major events have occurred because we ensure to buy good cattle from the auctions and meat is inspected by the Meat Inspector [LEOs or VOs] as required. If it has been established that a particular animal has infection and part of the meat has to be condemned, we cannot for sure influence the VO’s decision as it may cause a big problem to the customers.*

(Urban butchery)

*I think major events of that kind have not happened here because of the good quality of meat I buy every day. Even when the inspectors/visitors come they appreciate what I am doing. I cannot sell poor meat to customers. If in the process I come across such meat, I just throw it away. I want to retain and attract more customers and respect my work.*

(Urban butchery)

Overall, however, a majority of operators of butcheries tend to point to external agency when it came to explaining why there had been no food safety issues.

*This has never happened here because our meat inspectors are active and committed to their job.*

(Rural butchery)

*The meat is chopped hanged for inspection by the VO. After inspection, the VO stamps on the meat indicating that meat is safe to eat.*

(Rural butchery)

*We depend on meat inspectors to approve the safeness of the meat. After inspection they stamp the meat indicating that is safe to eat.*

(Urban butchery)

In marked contrast, people in urban and rural eateries mention their own preventative actions more often than external agents’ actions as explanations for why they have not experienced problems with meat safety (even though, as shown in the first quote, these actions may at times be guided by evidence of preceding external agency such as regulatory inspections.)

*I always buy inspected and stamped meat from the butcher. I don’t deal with uninspected meat as it may be very harmful to my clients.*

(Urban eatery)

*We clean every place; that is why no bad smell is coming from anywhere, you can feel fresh like you are on the beach. You may go and look to our washrooms they are clean, we are proud of this actually.*

(Rural eatery)

*This [problem with meat safety] has not happened because my boss is very keen. She ensures the meat we buy is fresh and is boiled for long time. If meat is well-cooked, I don’t expect it to cause any health problem when eaten.*

(Rural eatery)

Thus, a general trend is visible in which meat-sellers’ perspectives differ by occupation, with butchers relying, in part and more often, on external agency and eateries primarily emphasising their own agency. When asked about the future of meat safety risks, answers in both butcheries and eateries broaden out from their immediate workplace and reveal who and what they see as the main drivers of meat safety.

### 3.3. Expectations in Butcheries and Eateries Regarding Meat Safety in the Future

After exploring past experiences with problems in meat safety, each interviewee was specifically asked, “Do you think there will be any major problems regarding the food safety of meat in the future?” Quantitative analysis of their answers reveals two clear patterns. First, very few interviewees (varying between 6–13%) in any of the categories expect the status quo in meat safety to remain in the coming years. Large majorities in all categories expect significant changes (Table 2).

However, a second pattern is that interviewees in the urban environment are equally divided about the question whether problems with meat safety would decrease or increase in the future, as illustrated by the first two quotes below. In contrast, clear majorities of interviewees operating butcheries and eateries in a rural environment are more optimistic and expect meat safety problems to decrease, as the last two quotes show.

*More serious major events are going to occur in the future.*

(Urban butchery)

*The future is looking good; the trend is coming down.*

(Urban eatery)

*No major problem in future, actually I think they shall completely end.*

(Rural eatery)

*I think now most people are aware of this problem. As result, I believe in future the incidences will come down.*

(Rural butchery)

As with the earlier question on past experiences with problems with meat safety, most interviewees also volunteered, in an unprompted way, explanations for their expectations of the future of meat safety. They provided detailed assessments—both positive and negative—of a range of factors that they believed to be relevant. For example, one urban butcher who concluded that major diseases would disappear, did so because he believed that the presence of “many VOs” would reduce the risks by inspecting all meat, and when necessary condemning it. However, he was also aware that these official procedures of disease identification and meat condemnation may not entirely eliminate risk because “others may decide to eat it”.

When we analysed these explanations, we identified ten factors interviewees believed affected meat safety risks. The analysis of the five most frequently identified factors—namely veterinary and health inspection staffs’ practices (including VOs, LEOs and HOs), livestock owners’ practices, application of veterinary medication, butcheries’ professional practices and consumer preferences (see Table 3)—shows that the same factor can be a risk-increasing factor in the eyes of one interviewee, and a risk-decreasing factor in the eyes of another. These five leading factors are mentioned 49, 34, 22, 17 and 14 times, respectively, and are discussed in more detail below. The remaining five factors, mentioned between 12 and 3 times, refer to financial matters (ranging from “greed” to “too poor to buy drugs”); professional practices in eateries; wider drivers of change (e.g., climate change); or professional practices at auctions or slaughter sites, and can be seen to be of less relevance to meat safety.

The professional practices of veterinary staff and HOs are the most frequently mentioned factor affecting meat safety in the future (49 times). Table 3 shows most of these comments suggest interviewees believe this will reduce risks rather than increase risks (in a proportion of 30:19). It is clear that operators of butcheries and eateries consider the role played by VOs and LEOs as the most important factor affecting meat safety. This includes VOs’ responsibility for keeping livestock fit and healthy through vaccinations, slaughter inspections, advice, fines for transgressions, and certification, in the form of a blue ink stamp, that meat is safe to eat:

*VOs … These are very key people in the society.*

(Urban eatery)

What matters for operators of butcheries and eateries are the numbers of Veterinary staff or HOs, their availability, what these officers do and how they do it. Operators of butcheries and eateries who felt that meat safety problems would decrease generally also felt that these posts were adequately staffed:

*The problems will not increase because now we have many VOs, yes they are many … There are two inspectors here.*

(Urban butchery)

Furthermore, and in support of the impression that most of the comments reflected a positive view of the role of veterinary and health staff, those who felt that meat safety risks would persist or increase also explained that there were “not enough” of these officers and that they were frequently unavailable.

*If you need an emergency veterinary service now, you will never access it unless you have the mobile number of the officer concerned.*

(Rural eatery)

Nonetheless, it should be noted that comments by operators of eateries in both urban and rural areas seemed rather evenly divided in their assessment of the role and performance of veterinary and health officials, and some butchers were also critical, as shown in the following quotes.

*They know these eateries but they always ignore them because the owners are uncles or children. I also suspect that the law overseers receive bribes during inspection.*

(Rural eatery)

*You will see them writing reports, they are fake reports cooked up at the office desks. They don’t visit farmers and livestock keepers.*

(Urban butchery)

Livestock owners’ practices were the next most frequently identified factor affecting meat safety in the future. Most of the comments reflected the opinion that livestock owners were ignorant of, or ignored, animal healthcare (such as vaccinations), and that this will, in future, increase meat safety risks.

*You know that some livestock owners are very ignorant.*

(Urban butchery)

However, some comments suggested views that pastoralist livestock producers had good animal health knowledge, as the following quote shows:

*Even the Maasai themselves are aware of the importance of treating and vaccinating their animals.*

(Urban butchery)

In some instances, livestock producers’ knowledge of animal health was identified as increasing meat safety risks as skilled livestock owners were believed to knowingly deceive other meat value chain actors. Examples of deliberate deception include selling condemned meat and/or selling animals under medication without disclosing this, as the following quotes show.

*Some livestock owners sell their animals while under medication. This is ridiculous, as they understand it will affect consumers negatively.*

(Urban butchery)

*Animal vaccines and treatment are known to every livestock keeper and if a Livestock Inspector tells you that this animal is sick, you must believe it … The inspector may condemn a cow but after he [the inspector] has left, the meat is sold. This type of meat is usually sold secretly, at lower price.*

(Rural butchery)

The third most common factor mentioned as a driver of meat safety in the future is the use of veterinary medication. Most of the comments on “medication” (both treatment and vaccination) saw veterinary medication as a factor that reduced meat safety risks.

*I think the problems of food safety of meat will come down. For example, it is quite a long time now I have not heard of tapeworms anymore, it seems to have disappeared, and this is improvement. Better vaccination and treatment of animals.*

(Rural butchery)

However, there were also some comments expressing concerns about the increased availability and use of drugs—often described in terms of excessive or inappropriate dosages. These interveiwees were commenting in particular on the effect these could have on people eating the meat.

*If cattle have been injected with growth hormones to accelerate fattening of the animal, don’t you see that could cause problems to meat consumers?*

(Urban eatery)

The fourth factor identified in Table 3 are butchers’ practices in relation to meat safety in the future. There were 17 interviewees (ten in butcheries, seven in eateries) who volunteered comments on the practices in butcheries affecting meat safety. Most of them saw these practices as increasing meat safety risks; this involved deliberate deception (mixing fresh or inspected meat with old or uninspected) and ignorance or a lack of instruction as causes of an increase in meat safety risks. The following two quotes illustrate both issues.

*We see bad butchers slaughter cattle at home and mix the meat with the one at the butchery. The meat is not inspected.*

(Urban eatery)

*Sometime problems may occur because of negligence.*

(Rural butchery)

However, six interviewees believed that butcheries were improving their practices, as demonstrated through their increasing awareness and willingness to work with veterinary and health inspection staff, to undertake training and to comply with meat safety standards.

*We sometimes attend training on how to keep our business site clean. They tell us that we must clean all stains of blood at the slab because flies may pick and spread harmful bacteria to the rest of the meat at the butcher that will finally affect customers.*

(Urban butchery)

Some operators of butcheries and eateries recognised that reducing meat safety risks was not just a matter of compliance, it was also a means to increase their customers’ trust in them as sellers of good quality meat.

*The butcher men are supposed to buy healthy and unaffected animals. They must be very keen, not us, we are buying meat from them.*

(Rural eatery)

*We can participate as butchers by ensuring that we buy and sell safe meat to our customers. Consumers should also make sure that they buy and eat safe meat from genuine butchers.*

(Urban butchery)

This concern with providing consumers with safe meat is further connected with consumers’ preferences, the fifth-ranking factor identified as affecting meat safety in the future. There were 14 interviewees who talked about the importance of consumer preferences affecting meat safety, with most of them (nine) arguing that some consumers’ preferences for meat that was not quite well-cooked increased meat safety risks.

*You see we have some different types of customers, others prefer well-cooked meat, others just cooked meat. Under such a situation what can we do? We just follow their instructions. If the meat has harmful bacteria, these customers will be affected.*

(Urban eatery)

Hitherto in this section, the analysis of data presented in Table 3 focused on the five principal factors that interviewees identified as increasing or reducing meat safety risks. A further analysis of the totalled risk assessments of these factors by business type and location provides additional in-depth insight in how risk assessments correlate with types of businesses and location. Two particular patterns come to the fore.

First, when disaggregating by urban or rural location, the total number of responses expecting food safety risks to reduce comprise a majority of the rural meat-sellers’ comments in a 41:26 ratio (27 + 14:10 + 16). The risk assessments by their urban counterparts tend in the opposite direction in a very similar but inverted proportion of 28:41 (20 + 8:19 + 22). This pattern in the frequency of risk assessments seems to align with the earlier finding about interviewees’ general expectations about meat safety in the future, which also showed rural operators are more optimistic in expecting meat safety problems to decline (Table 2). Whereas urban operators of both business types were rather evenly balanced when asked about their general expectations (Table 2), the findings in Table 3 show that urban operators do list more specific factors that may increase risk.

A second pattern emerging from Table 3 suggests that, within the urban or rural locations, there are clear differences between the risk assessments by business type. The high number of more pessimistic risk assessments among urban operators (22:8) is largely made by operators of urban eateries; comments by urban butchers are more evenly balanced in a 19:20 ratio. Remarkably, the high number of more optimistic risk assessments among rural interviewees is largely provided by rural butchers (10:27); comments by operators of rural eateries are rather balanced in a 16:14 ratio. This analysis of the data from Table 3 suggests the overall expectations regarding future meat safety tends to be more optimistic in rural areas but is largely carried by rural butchers. The more pessimistic outlook emerging from urban areas is, in contrast, mostly based on risk assessments by the operators of urban eateries.

### 3.4. Strengths and Limitations

One particular strength of this research is that is it captures patterns and recurring issues in the personal views of people who, because they handle raw and cooked meat just before it reaches consumers, are directly affecting meat safety. They represent some of the last links in the meat value chain. Most importantly, the presented data reflects their views on meat safety and meat safety regulation and enforcement in their words. While this is an important perspective in understanding meat safety risks, there is also a limitation of this approach; it does not explicitly capture the risk caused by invisible pathogens that may be present in the gut of healthy animals such as non-typhoidal *Salmonella* and *Campylobacter*, which is the microbiological focus of the HAZEL project. The findings bear additional relevance, exemplified through the disaggregated comparative presentation of data on both a rural-urban divide as well as a divide between the two business types. Another strength is that the sample of 64 interviewees is rather large in qualitative research terms and, in the case of the butcheries, is fairly representative covering about 5% of the existing businesses. On the other hand, one of the limitations of the research is that the semi-structured character of the interviews provides responses that reflect what interviewees were willing to share or had knowledge of and may not reflect daily practice. It is, of course, entirely possible a butchery or eatery could be involved in a major meat safety event without being aware of it. Another limitation may be a potential selection bias of interviewees within a randomly selected ward. Interviewees were selected by local authorities, as required per Tanzanian protocol. However, we do not believe that this stifled dissident views; authorities were not present during the (anonymous) interviews and interviewees shared a range of views about local authorities—which were not always positive.

## 4. Discussion

Two key themes emerge from the above results. The first concerns the way in which raw and cooked meat-sellers’ conceptualise their agency in relation to meat safety. The second involves their perceptions of meat safety risks in the future and the extent to which these will increase or decrease.

### 4.1. Business Type and Agency Within the Meat Value Chain

Moshi’s meat-sellers articulated different degrees of agency in relation to meat safety. As shown in the data above, butchers, both urban and rural, saw their ability to ensure meat safety as relatively limited and emphasise the role of regulation, inspection, and other structural procedures. They emphasised, in particular, the work of veterinary staff. These government actors form part of “an extensive network of district-level, government sponsored, livestock extension services and limited private practice” [52] (p. 2). Part of their role is to ensure that cattle bought, slaughtered and sold by butchers is safe to eat. In this regard, VOs and LEOs offer services to livestock owners, who can request and pay for treatment for sick livestock. They provide vaccination—which may be free if it accords with national or donor priorities—and they advise on “withdrawal periods” after treating livestock with antibiotic medication [52] (p. 2). VOs and LEOs play an important role in district-level disease surveillance, reporting disease incidents (based on passive surveillance and clinical diagnosis) to zonal veterinary investigation centres [2].

These government officers are also frequent visitors at the butchers’ shops and slaughter facilities, undertaking pre- and post-mortem inspection, condemning diseased organs and, as indicated in some of the above quotes, stamping the animal carcass in several locations. This highly-visible stamp assures customers that the meat has been checked. All these actions help butchers communicate that their meat is safe for human consumption. Both urban and rural butchers placed considerable faith in these acts of inspection. While this role is of significance in reassuring consumers through a visible marker that butcheries are supervised by authorities, inspection of meat itself is not the only way in which meat safety is ensured and the VOs and LEOs—and indeed other state actors such as Hos—also play an important role making sure that butchers comply with regulations such as using the mandated equipment (modern meat saws, plastic chopping boards), having the required infrastructure (tiled walls, screens, etc.) and meeting health and hygiene standards (overalls, cleanliness, etc.). There is a degree of irony in the finding that a majority of operators of butcheries mention external agency more often as an explanation for food safety than their own agency because the inspectors’ role is to ensure that butchers comply with regulations and act as responsible agents in the provision of safe meat—in other words, that butchers themselves demonstrate agency for meat safety. (We also researched the actual enforcement of regulations regarding health and hygiene standards in Moshi Urban and Moshi Rural [21].)

Such perceptions of limited agency contrast with operators of eateries who strongly emphasised their own agency in relation to meat safety. Other research finds that Tanzanian slaughter workers experience less agency than butchers ([47] under review). This suggests that actors’ subjective experience of control increases across the value chain, with slaughter workers feeling least empowered to ensure meat safety, butcherers having some sense of agency and finally eatery operators feeling that they are able to ensure meat safety through their own actions. Indeed, respondents from eateries stressed their roles in cleaning their facilities and equipment, in ensuring that only inspected, stamped and quality meat was purchased, and in cooking meat well to ensure its safety. While eateries, like butcheries, are also inspected in Tanzania (by HOs rather than VOs or LEOs), inspection is less frequent and focused on ensuring that the conditions under which the food is prepared comply with regulations. In fact, Tanzania’s regulations do not stipulate any minimum frequency for inspections by HOs and leave it explicitly to the local authorities to “set the frequency of inspection” [53] (p. 6). Our conversations with local authorities in Moshi Urban and Moshi Rural suggest the number of HOs at local level is very low in regard to the number of locations they could inspect and decisions about inspection visits are largely driven by urgent issues and the availability of transport or fuel. One source suggested visits may be monthly for some locations and quarterly for many others.

In theory, however, all raw meat in butcheries is officially inspected and stamped before it goes out for sale to consumers, whereas cooked meat in eateries goes to consumers without an official inspection. Butchers see inspectors every time they butcher an animal, which may be several times a week, or even daily. People working in eateries see inspectors only periodically when their business premises’ compliance with regulations is checked, but the meals they serve are not inspected. This is likely to explain to some extent why people working in eateries emphasise their own actions as contributing to meat safety, rather than inspectors’ actions.

Moreover, the sense of agency among operators of eateries may also stem in part from their regular and immediate interactions with customers consuming meat that they have prepared. As such, they have less scope than butchers, who are removed from the cooking and consumption of meat, to deny possible cases of illness stemming from meat they have sold. In addition, prevailing local understanding about meat safety is directly associated with the practice of cooking meat for extended periods of time. In contrast, the activities which butchers undertake are not directly implicated in local narratives of food safety and work they do to limit the spread of pathogens is less recognised. Thus, eatery operators repeatedly stressed the importance of, and their efforts in, cooking meat well in order to ensure its safety.

### 4.2. A Rural-Urban Divide over the Future of Meat Safety

Food safety issues in Tanzania are predicted to increase in the future in conjunction with economic growth and increased expenditure on animal-sourced food among Tanzania’s rapidly growing and urbanising population [41]. Red meat consumption is projected to increase by 87% by 2030 [27,40]. Coupled with intensified production systems, centralised production and wider distribution networks—all of which are required to support the demands of growing and urbanising populations—this potentially places many people at risk of pathogen exposure from the same source. As a consequence, foodborne disease incidents are likely to increase in frequency and to command increasing political attention [41].

While we have found a clear difference between operators of butcheries and operators of eateries in terms of self-perceived ability to ensure meat safety and a corresponding difference between their relative senses of agency, when it comes to interviewees’ expectations for meat safety in the future, the divide seems to exist between urban and rural meat-sellers. Rural meat-sellers’ optimism about meat safety in the future seems largely supported by rural butchers, whereas the more pessimistic outlook among urban meat-sellers seems driven more by the operators of urban eateries who predict that risks will increase. Taken together, these patterns raise the question of why rural meat-sellers—particularly rural butchers who seem to have a lower sense of the importance of their own agency when it comes to ensuring meat safety than operators of rural eateries—seem so positive about meat safety in the future.

Food risk perceptions can be affected by people’s residence in urban or rural localities. For example, Prain and colleagues argue that, in Kampala, Uganda, “farming systems intensify as they get closer to urban centres with their demands for food and their lack of space. Perceptions of health risks associated with agriculture and the elaboration of mitigation strategies also seem to intensify” [54] (p. 240). Rural-urban divergences in risk perceptions may also be underpinned by consumers’ diets, education, income and other factors, with younger, more skilled urban consumers worrying about food quality and diversity while rural residents emphasise cost and availability [41]. Similarly, it might be expected that geographical distance and the nature of the value chain relationships affect Tanzanian butchers’ risk perceptions, with urban meat-sellers more concerned about meat safety. In general, Tanzania’s rural butcheries tend to purchase directly from livestock producers at rural markets and butchers are likely to know and interact with the livestock owners.

Moreover, rural butchers are likely to be personally familiar with local cattle rearing practices. In contrast, urban butchers buy cattle at resale auction markets and generally do not interact with the livestock owners who raised these cattle. The urban meat value chain is quite long and intermediated by diverse parties, from rural livestock producers, rural auction markets, wholesale traders who transport live cattle, urban resale markets, abattoirs or slaughter houses, and finally butcheries and/or eateries [55,56,57]. As Ladbury has argued, in relation to milk value chains in northern Tanzania, trust is a fundamental part of value chain governance and “wherever possible, value chain actors would choose to do business only with actors whom they believed they could trust” [58] (p. 151). This suggests that rural meat-sellers (especially butcheries), who live in close proximity to the producers and have shorter value chains, experience greater optimism about food safety than do urban meat-sellers, who experience significant geographical distance from the producers and longer, intermediated, value chain relationships and who are more ambivalent about the future (see Table 2).

The idea that geographical distance and the nature of value chain relationships affect risk perceptions, is evident in the divergent views of urban and rural meat-sellers regarding meat safety in the future. The optimism of rural butchers seems predicated on the fact that a majority of the rural meat-sellers’ comments on the practices of veterinary and health officials—the most commented upon risk factor—are positive, trusting these practices will improve and expand and thereby reduce meat safety risks. This optimism may however be somewhat misplaced. Tanzania’s Livestock Master Plan of 2018 officially identifies, among other matters, an inadequate supply and inefficacy of veterinary pharmaceuticals as core challenges [40]. Other research underscores that veterinary drugs are predominantly supplied by private vendors and that, compared to urban areas, rural areas have less access to these private veterinary staff and pharmaceuticals. This bias favouring urban and peri-urban areas also goes for public veterinary services [52,55]. Moreover, more recent research reconfirms veterinary drugs are rarely available in rural areas and, when they are, may be uncontrolled, substandard and falsified drugs sold without skilled veterinary support [59]. While the Tanzania government attempts to improve the situation with policies paying more attention to vaccination, cattle dips, and veterinary drugs [60], effects in rural areas may only become visible in the medium term. In all, these conditions in rural areas do not warrant the optimism regarding meat safety that we found among the majority in rural meat-sellers, yet their proximity to—and knowledge of—producers and shorter value chains may lead them to take a more positive view on food safety.

## 5. Conclusions

The literature on red meat value chains in Tanzania recognises the importance of food safety [40,61] and appreciates that this is “a widely shared responsibility that requires positive interaction between all stakeholders” [62] (p. 9). While there is growing body of research on food safety in butcheries and eateries [20,22,63,64], much of it focuses on economic and health aspects and little attention has been paid to the ways in which operators of eateries selling cooked meat and butchers selling raw meat see their own and others’ agency in reducing food safety risks. Findings from our research among butcheries and eateries in northern Tanzania reveal two striking patterns. First, operators of eateries see their agency in meat-handling practices as critically important to maintain meat safety, much more than butchers selling raw meat. The latter place considerable trust in inspectors—who inspect and stamp every butchered animal prior to sale to consumers—for ensuring that the raw meat is safe for consumption. Arguably, the much more regular and product-focused official inspections of butcheries reduce the sense of agency and responsibility for meat safety among butchers, while the more periodic and premises-focused inspections of eateries are likely to contribute to sense of agency and responsibility in eateries. Quite possibly this is reinforced by cultural perceptions which emphasise that meat safety is predicated on what live animals and raw meat look like when purchased and the way meat is cooked and served rather than butchered and stored.

Our second finding is equally striking: namely rural meat-sellers, particularly rural butchers, are clearly more optimistic than their urban counterparts that meat safety will improve in the future. Rural meat-sellers operate in shorter, relatively unmediated value chains, and in closer proximity to both livestock producers and consumers. Thus, despite rural areas having less access to veterinary and health services, and rural meat-sellers having fewer inspections [59,65], they are more optimistic about the future. This is not, as our research shows, influenced by meat-sellers’ past experiences and socio-cultural contexts [44,66]; rather it is the nature of the value chain and their close proximity to livestock producers which drives this optimism.

In terms of recommendations for local authorities and further research, we have four observations. First, local authorities enforcing policies in a resource-poor context may explore the potential for more efficient or even solicited inspections, by building on the finding that meat inspections add commercial value—particularly meat sold in butcheries. Further research should assist local authorities to cooperate with butcheries and identify ways to organise this better [21]. Similarly, further research could explore the risk embodied in invisible pathogens, and to what extent visible signs of regular inspections, such as stamps, may have commercial value for meals cooked in eateries.

Second, and necessarily in conjunction with the first recommendation, local authorities and researchers need to carefully investigate further, and consider the consequences of, the finding that (increased) inspections seem to decrease the sense of agency (and possibly responsibility) regarding meat safety of the operators of inspected eateries and butcheries. Operators may welcome inspections as it adds value to their product, but unless inspections are also clearly contributing to better food handling practices, public health benefits are likely to remain elusive or even deteriorate.

Third, the preoccupation of inspection with visible abnormalities suggest a lack of awareness around invisible pathogens originating from healthy animals’ gastrointestinal tracts ([47] under review). The microbiological component of HAZEL suggests there is an opportunity for butchers in particular to take responsibility for avoiding contamination of meat with such pathogens.

Finally, our findings suggest rural operators and urban operators may, quite possibly, respond differently to policy interventions because of their different expectations of the future. Further research is needed to determine how these diverging expectations may affect operators’ response to any such future-oriented interventions.

## Figures and Tables

**Table 1:** Number of times agency was mentioned to explain the prevention of major events with meat safety ^1) 2) 3).^

	Urban		Rural	
	External Agency	Own Agency	External Agency	Own Agency
Butcheries	8	7	11	6
Eateries	4	12	2	11
Total	12	19	13	17

^1)^ Urban butcheries, n = 17; rural butcheries, n = 15; urban eateries, n = 17; rural eateries, n = 15; total (T) = 64. ^2)^ Note: 53 of the 64 interviewees (83%) volunteered explanatory views. ^3)^ Interviewees could volunteer to mention either only external agency (e.g., inspectors, regulations), or only their own agency (e.g., cleanliness, selection, preparation), or mention both external and own agency as an explanation why there had been no major events with meat safety.

**Table 2:** Expectations regarding major problems with meat safety in the future ^1)^.

	Urban				Rural				
	Problems will increase	Problems will decrease	Problems remain same	Don’t know n/a		Problems will increase	Problems will decrease	Problems remain same	Don’t know n/a
Butcheries	35%	41%	6%	18%		20%	73%	7%	-
Eateries	47%	41%	-	12%		27%	60%	13%	-

^1)^ Urban butcheries, n = 17; rural butcheries, n = 15; urban eateries, n = 17; rural eateries, n = 15; total (T) = 64.

**Table 3:** Risk assessment of factors that operators of butcheries and eateries believe affect future meat safety risks and the number of times they are cited across business types and locations ^1) 2).^

	Urban				Rural				Total
	Butcheries		Eateries		Butcheries		Eateries		
Factors	Increase risks ↑	Reduce risks ↓	Increase risks ↑	Reduce risks ↓	Increase risks ↑	Reduce risks ↓	Increase risks ↑	Reduce risks ↓	
1. Veterinary/ Health staff practices (T = 49)	4	9	7	6	3	11	5	4	↑ = 19 ↓ = 30
2. Livestock owners’ practices (T = 34)	6	4	5	-	4	6	6	3	↑ = 21 ↓ = 13
3. Veterinary Medication (T = 22)	1	3	3	2	1	7	2	3	↑ = 7 ↓ = 15
4. Butchery practices (T = 17)	4	2	3	-	2	2	2	2	↑ = 11 ↓ = 6
5. Consumer preferences (T = 14)	4	2	4	-	-	1	1	2	↑ = 9 ↓ = 5
Total	19	20	22	8	10	27	16	14	136

^1)^ Urban butcheries, n = 17; rural butcheries, n = 15; urban eateries, n = 17; rural eateries, n = 15; total (T) = 64. ^2)^ Note: 53 of the 64 interviewees (83%) volunteered the total of 136 assessments of these risk factors.
